# Evaluation of oxidative stress biomarkers in idiopathic pulmonary fibrosis and therapeutic applications: a systematic review

**DOI:** 10.1186/s12931-018-0754-7

**Published:** 2018-03-27

**Authors:** Alessandro G. Fois, Panagiotis Paliogiannis, Salvatore Sotgia, Arduino A. Mangoni, Elisabetta Zinellu, Pietro Pirina, Ciriaco Carru, Angelo Zinellu

**Affiliations:** 1 Department of Clinical and Experimental Medicine, University of Sassari, Sassari, Italy; 2 Department of Respiratory Diseases, University Hospital Sassari (AOU), Sassari, Italy; 3 Department of Biomedical Sciences, University of Sassari, Sassari, Italy; 4 Department of Clinical Pharmacology, College of Medicine and Public Health, Flinders University, Adelaide, Australia

**Keywords:** Lung, Idiopathic pulmonary fibrosis, Oxidative stress, Oxidant, Antioxidant

## Abstract

**Introduction:**

Idiopathic pulmonary fibrosis (IPF), a fatal lung disease of unknown origin, is characterized by chronic and progressive fibrosing interstitial pneumonia which progressively impairs lung function. Oxidative stress is one of the main pathogenic pathways in IPF. The aim of this systematic review was to describe the type of markers of oxidative stress identified in different biological specimens and the effects of antioxidant therapies in patients with IPF.

**Methods:**

We conducted a systematic search of publications listed in electronic databases (Pubmed, Web of Science, Scopus and Google Scholar) from inception to October 2017. Two investigators independently reviewed all identified articles to determine eligibility.

**Results:**

After a substantial proportion of the initially identified articles (*n* = 554) was excluded because they were duplicates, abstracts, irrelevant, or did not meet the selection criteria, we identified 30 studies. In each study, we critically appraised the type, site (systemic vs. local, e.g. breath, sputum, expired breath condensate, epithelial lining fluid, bronchoalveolar lavage, and lung tissue specimens), and method used for measuring the identified oxidative stress biomarkers. Furthermore, the current knowledge on antioxidant therapies in IPF was summarized.

**Conclusions:**

A number of markers of oxidative stress, with individual advantages and limitations, have been described in patients with IPF. Nevertheless, trials of antioxidant treatments have been unable to demonstrate consistent benefits, barring recent pharmacogenomics data suggesting different results in specific genotype subgroups of patients with IPF.

## Background

Idiopathic pulmonary fibrosis (IPF) is a fatal lung disease of unknown cause that is characterized by chronic and progressive fibrosing interstitial pneumonia with worsening dyspnea and lung function [1, 2]. Though IPF is relatively rare it is the most common and severe form of idiopathic interstitial pneumonia (IIP) [3]. Histologically, IPF is characterized as usual interstitial pneumonia (UIP), a form of disease with progressive fibrosis of the lungs [4, 5].

Risk factors for IPF include cigarette smoking, environmental factors, microbial agents, and gastroesophageal reflux [1, 2, 6]; recent studies also support the role of gene expression and epigenetic alterations [7, 8]. Most of these factors are also involved in other lung pathologies such as lung cancer [9, 10]. IPF symptoms include dry cough, dyspnoea, and digital clubbing [11]. Pulmonary function tests identify restrictive features (reduced total lung capacity) and abnormal gas exchange (reduced capacity for carbon monoxide diffusion) [1]. Diagnosis often requires a multidisciplinary approach and, in some cases, a lung biopsy [1, 12].

Although IPF has long been considered a chronic inflammatory disorder, this concept has been revisited following the negative results of interventional studies with anti-inflammatory therapies [13]. IPF is now widely accepted as a consequence of multiple interacting genetic and environmental risk factors, which cause repetitive local micro-injuries to ageing alveolar epithelium [11]. This triggers aberrant epithelial–fibroblast communication, induction of matrix-producing myofibroblasts, remodelling of the interstitium, and dysregulated repair of the injured lung [11].

There is growing evidence that oxidative stress plays a significant role in IPF [14, 15]. Oxidative stress is defined as an imbalance between oxidant production and antioxidant defence in favour of oxidants, that leads to cellular dysfunction and tissue damage. Due to its exposure to relatively higher oxygen tensions than other tissues, the lung is particularly sensitive to oxidative stress. Exogenous oxidants and pollutants further increase oxidant production and activate inflammatory cells to generate free radicals. Cigarette smoke, asbestos fibers, drugs and radiations, are well-known to favour fibrotic interstitial lung reactions [14]. Furthermore, they have been shown to trigger the production of the reactive oxygen species (ROS) hydroxyl radical, hydrogen peroxide, and superoxide radical. In the human lung, several pathways can generate ROS, including nicotinamide adenine dinucleotide phosphate oxidases, myeloperoxidase, eosinophil peroxidase, mitochondrial electron transport chain, and xanthine oxidase [16, 17]. In addition, superoxide may react with nitric oxide (NO) to form various reactive nitrogen species (RNS), such as peroxynitrite. NO is principally produced by the inducible form of nitric oxide synthase (iNOS, NOS2) in the lung, in particular during inflammation. Moreover, human lung cells widely express also the constitutive forms of NOS, that further contribute to NO production. In general, a complex variety of oxidants are produced in response to injuries leading to pulmonary fibrosis. These oxidants can activate several genes related to cell growth, cell death, and fibroblast proliferation [1].

Normal pulmonary homeostasis requires an appropriate balance between intracellular and extracellular oxidants and antioxidants. Lung protection against oxidants is guaranteed by protective antioxidants and antioxidant enzymes that include (i) small-molecular-weight antioxidants (e.g., glutathione, vitamins, uric acid), (ii) mucins, (iii) metal-binding proteins (transferrin, lactoferrin, metallothionein, etc.), (iv) intracellular and extracellular superoxide dismutases (SODs), (v) enzymes to reduce H_2_O_2_ (several glutathione-associated enzymes and catalase), (vi) detoxification enzyme systems (e.g., glutathione-S-transferases), and (vii) other redox regulatory thiol proteins (e.g., thioredoxin-peroxiredoxin system and glutaredoxins) [16–19].

**Fig. 1:**
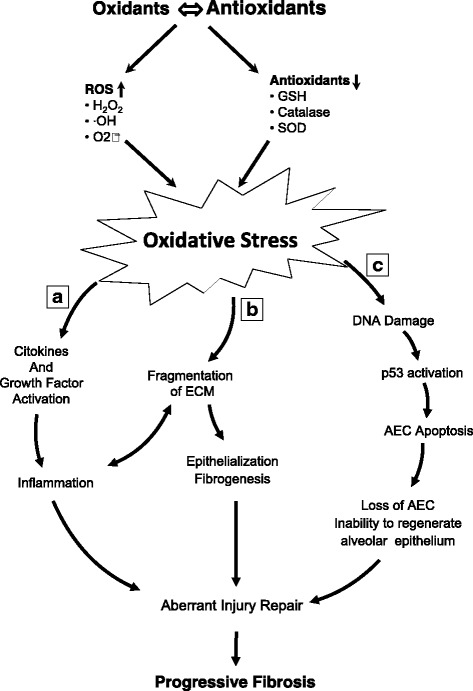
Oxidative stress plays a critical role in the pathogenesis of IPF through: **a**) promoting inflammation by increasing production of cytokines and growth factor, which causes **b**) fragmentation of extracellular matrix, increased myofibroblastic differentiation, fibrogenesis and epithelialization; **c**) as a consequence of DNA damage and p53 activation, ROS promote apoptosis of airway epithelial cells resulting in an impaired ability to regenerate alveolar epithelium. These alterations contribute to the progression of pulmonary fibrosis

These majority of these enzymes, localized in bronchial and alveolar epithelial cells, alveolar macrophages, and the extracellular milieu, are regulated by the nuclear factor erythroid-derived 2-like2 protein (Nrf2), which controls the expression of several antioxidant pulmonary proteins. The importance of Nrf2 in IPF has been demonstrated through experiments in mice in which deficiency of this transcription factor significantly enhanced bleomycin-induced pulmonary fibrosis [20]. It is likely that the induction of antioxidant enzymes and related proteins after exposure to insults may protect the lung and promote damage repair. Conversely, reduced induction or inactivation of antioxidant enzymes may result in a continuous redox imbalance, that may contribute to the progression of pulmonary fibrosis (Fig. 1).

Given the accepted role of oxidative stress in IPF, the aim of the present systematic review was to critically assess published studies investigating the type of oxidative stress markers in different biological specimens, the advantages and limitations of the methods used for their measurement, and potential therapeutic implications in this patient group.

## Search strategy and study selection

**Fig. 2:**
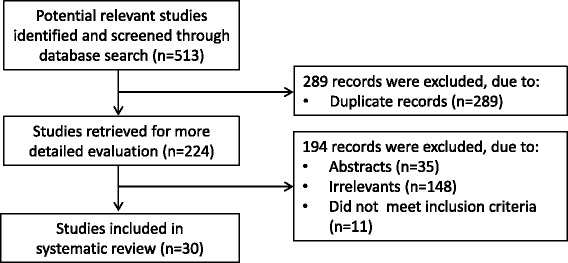
Flow chart showing the literature search and selection. Specific reasons for exclusion of studies are also shown

A systematic search of publications listed in electronic databases (Pubmed, Web of Science, Scopus and Google Scholar) from inception to October 2017, was conducted using the following terms: “oxidative stress”, “IPF”, “idiopathic pulmonary fibrosis” as well as combinations of these terms. Two investigators independently reviewed the identified articles to determine their eligibility. Studies were considered eligible if they met the following criteria: (1) assessment of oxidative stress (OS) biomarkers in any type of biological specimens from IPF patients; and (2) English language full-text publications involving humans in peer reviewed journals. Abstracts were screened independently and, if relevant, full articles were obtained and reviewed. References in the retrieved articles were also reviewed to identify additional studies. A flow chart showing the study selection is presented in Fig. 2.

From a total of 513 initially identified studies, 289 were excluded after the first screening, mainly because they were duplicates. The majority of the remaining 224 studies were also excluded, mainly because they were either in abstract format, irrelevant, or did not met the selection criteria. A total of 30 studies were included in the systematic review.

## Biomarkers of oxidative stress in IPF

The identification of oxidative stress biomarkers in IPF patients was performed in different biological specimens, primarily blood and serum (*n* = 10 studies), bronchoalveolar lavage fluid (BALF, *n* = 10), and lung tissue (*n* = 9), with relatively few studies using sputum (*n* = 1), breath (*n* = 1), epithelial lining fluid (ELF) (*n* = 4), and expired breath condensate (EBC) (*n* = 4), (Table 1).

**Table 1:** Summary of the oxidative stress biomarkers studied in different specimens of IPF patients

Biological specimens	Comparison (n)	Oxidative stress biomarkers	Ref.
Plasma	IPF (36) vs Ctrl (31)	TEAC↓; PSH↔; MDA↑	21
Plasma	IPF/UIP (10) vs Ctrl (10)	Hydroperoxides↑	22
Plasma and urine	IPF (29) vs Ctrl (6)	Pl-isoprostanes↑; Ur-H_2_O_2_↔	24
Plasma and urine	IPF at rest (29) vs IPF after physical exercise (29)	Pl-isoprostanes↔; Pl-TAC↓; Ur-isoprostanes↑; Ur-H_2_O_2_↔	24
Serum	IPF (16) vs Ctrl (17)	8-Isoprostane↑	25
Blood	IPF (22) vs Ctrl (29)	GSH↓; GSSG↑; GSH/GSSG↓	26
Serum	IPF (43) vs Ctrl (30)	Hydroperoxides↑	27
Serum	AE-IPF (13) vs IPF (30)	Hydroperoxides↑	27
Plasma	IIP (31-9IPF) vs Ctrl (33)	TOS↑; OSI↑; TAS↔	28
Blood and plasma	IPF (10) vs Ctrl (9)	Bl-GSH↓;Bl-GSSG↔; Bl-Vitamin C↔ Pl-Uric acid↔; Pl-TEAC↓	29
Breath	ILD (34-13IPF) vs Ctrl (9)	Ethane ↑	30
Sputum	IPF (16) vs Ctrl (15)	GSH↓	31
EBC	IPF (16) vs Ctrl (15)	H_2_O_2_↑; 8-isoprostane↑	33
EBC	IPF (38) vs Ctrl (14)	MDA↔	34
EBC	IPF (20) vs Ctrl (20)	3NT↑; NOx↔; Proteins↑; 8-isoprostane↑;H_2_O_2_↔;	35
EBC	IPF (6) vs lung cancer (6)	8-isoPGF_2α_↑	36
ELF	IPF (44) vs Ctrl (11)	MPO↑	42
ELF	IPF (15) vs Ctrl (19)	tGSH↓; GSH/(GSH + GSSG) ↔	46
ELF	IPF (10) vs Ctrl (10)	tGSH↓; rGSH↓	49
ELF	IPF (17) vs Ctrl (14)	tGSH↓	50
BALF	IPF smokers (8) vs Ctrl smokers (6)	Protein carbonyls↔	52
BALF	IPF nonsmokers (9) vs Ctrl nonsmokers (14)	Protein carbonyls↑	52
BALF	IPF (9) vs Ctrl (5)	Protein carbonyls↑	54
BALF	IPF (13) vs Ctrl (5)	Protein carbonyls↑	55
BALF	IPF (15) vs Ctrl (8)	Protein carbonyls↑	56
BALF	s-IPF (17) vs f-IPF (10)	isocitrate dehydrogenase↓; peroxiredoxin 1↓; antithrombin III↓; complement factor B↓	57
BALF	IPF (17) vs Ctrl (14)	tGSH↔	50
BALF	IPF (36) vs Ctrl (31)	TEAC↓; GSH↓; GSSG↔; PSH↓; MDA↑	21
BALF	IPF (17) vs Ctrl (27)	GSH↔; Uric Acid↑; Ascorbic Acid↑; α-tocopherol↑; retinol↑; GSSG↑; F2-isoprostanes↔	58
BALF	IPF (16) vs Sarcoidosis (55)	8-isoprostane↓	25
Lung tissue	IPF (10) vs Ctrl (10)	ECSOD↔	59
Lung tissue	IPF fibrotic areas vs IPF normal areas	ECSOD↓	59
Lung tissue	IPF (10) vs Ctrl (10)	PrxII ↔	60
Lung tissue	IPF fibrotic areas vs IPF normal areas	PrxII↓; 3-NT↓	60
Lung tissue	IPF (7) vs Ctrl (7)	NRF2↔; SRX1↑	61
Lung tissue	IPF hyperplastic epithelium vs IPF normal epithelium	NRF2↑; KEAP1↑	61
Lung tissue	IPF (14) vs Ctrl (10)	GST ↓; Hp↓	63
Lung tissue	IPF (14) vs fNSIP (8)	PRDX6↓; TPxB↓;	63
Lung tissue	IPF (13) vs Ctrl (15)	Cys↑; Gly↑; Glu↑	64

### Systemic blood biomarkers of OS in IPF

Unlike EBC, sputum or breath, the measurement of OS biomarkers in blood has significant advantages in terms of reproducibility and sensitivity, but also challenges regarding their biological and clinical relevance when compared to local concentrations. Rahman et al. [21] measured parameters of oxidant/antioxidant balance in the plasma of 12 patients with IPF (7 non-smokers and 5 smokers) and 31 healthy subjects (23 non-smokers and 8 smokers). Plasma TEAC (trolox equivalent antioxidant capacity) was significantly lower both in healthy smokers and in patients with IPF regardless of their smoking status, when compared to healthy non-smokers. By contrast, there were no significant differences in plasma protein thiol concentrations between patients with IPF and healthy subjects.

Plasma concentrations of malondialdehyde (MDA) were higher in healthy smokers than in non-smokers, however they were similar between IPF patients who smoked and healthy smokers. The concentrations of products of lipid peroxidation were higher in patients with IPF, regardless of their smoking status, when compared to healthy non-smokers. These findings indicated for the first time that in IPF local oxidative stress is accompanied by a significant imbalance in systemic redox status.

Daniil et al. [22] evaluated plasma oxidative stress by measuring total hydroperoxides using a spectrophotometric method named D-ROMs [23]. Serum concentrations of hydroperoxides in IPF patients were significantly higher than controls (356 ± 14 vs 201 ± 10 UCarr, *p* < 0.001). No significant differences were observed between ex-smokers and never smokers. In the IPF group, there was a significant positive correlation between serum concentrations of hydroperoxides and severity of dyspnea, according to the Medical Research Council (MRC) chronic dyspnea score. Moreover, there was a negative correlation between the serum concentrations of hydroperoxides, forced vital capacity (FVC), and the diffusing capacity of carbon monoxide (DLCO). Nevertheless, no significant correlations were observed between oxidative stress parameters and Forced Expiratory Volume in the 1st second (FEV_1_), FEV_1_/FVC ratio, PaO_2_, and PaCO_2_. The authors suggested that the measurement of hydroperoxides concentrations might be useful in the clinical assessment of patients with IPF.

Jackson et al. [24] measured isoprostanes, total antioxidant capacity (TAC) and H_2_O_2_ in plasma and urine samples of 29 IPF patients and six healthy controls. IPF patients also underwent a standardized bicycle exercise protocol to quantify dyspnea. IPF plasma isoprostane values did not change significantly after exercise, but they clearly exceeded the normal control range for the assay. By contrast, IPF urine isoprostane concentrations increased significantly after exercise. Moreover, plasma TAC decreased significantly after exercise, along with the increment of lactate concentrations and hypoxemia, indicating increased systemic oxidant stress. There were no statistical differences in H_2_O_2_ urine concentrations between IPF patients, both at baseline and after exercise, and healthy subjects. The authors concluded that IPF patients have evidence of systemic oxidant stress at rest, and that low-level exercise produces reactive oxygen species in this group.

Similarly, Malli et al. [25] observed higher serum concentrations of 8-isoprostane in 16 IPF patients when compared to 17 healthy controls [median 77.25 pg/ml (IQR 52.42–162.5 pg/ml) vs 18.88 pg/ml (IQR 15.90–32.73 pg/ml)]. Muramatsu et al. [26] retrospectively evaluated the effect of inhaled N-acetylcysteine (NAC) monotherapy on redox balance and lung function, in 22 patients with early untreated IPF and 29 controls, in one of the few studies published which evaluated OS biomarkers in antioxidant therapies. FVC, blood concentrations of total glutathione (tGSH) and oxidized glutathione (GSSG), as well as the tGSH/GSSG ratio, and urine concentrations of 8-hydroxy-2′-deoxyguanosine (8OHdG) were measured at baseline, 6 and 12 months after initiating the treatment. The patients were divided in two groups according to the response to treatment at six months: patients with disease progression (FVC decrease of ≥5%), and patients with stable disease (FVC decrease of < 5%). Baseline tGSH concentrations and tGSH/GSSG ratio were both significantly lower in IPF patients than in healthy controls, while baseline GSSG concentrations in IPF patients were significantly higher than those in controls. Mean temporal changes in tGSH in patients with stable disease were similar to those in patients with progressive disease. However, baseline and follow-up GSSG concentrations in patients with stable disease were significantly higher than patients with progressive disease. The baseline tGSH/GSSG ratio was significantly lower in patients with stable disease when compared to patients with progressive disease, and the mean change during the follow-up was variable. Moreover, there was a significant negative correlation between the change in GSSG and the change in FVC, and a significant positive correlation between the change in tGSH/GSSG ratio and the change in FVC.

Finally, ROC curves analysis showed that GSSG baseline concentrations ≥1.579 μmol/L yielded the best sensitivity (69%) and specificity (100%) in discriminating between stable and progressive disease. The authors concluded that baseline GSSG concentrations may predict the efficacy of inhaled NAC for IPF, and that early therapeutic intervention with a GSH precursor may delay IPF progression, especially in patients with higher pre-treatment whole blood GSSG concentrations.

Matsuzawa et al. [27] measured serum hydroperoxide concentrations in 43 treatment-naïve IPF patients and 30 healthy controls, using d-ROMs. Among the 43 IPF patients, 27 had been followed-up for six months; furthermore, hydroperoxide concentrations were measured, before treatment, in 13 cases with acute exacerbation. Hydroperoxide concentrations in IPF patients were significantly higher than those in controls [median 366 U.CARR (IQR 339–443) vs. 289 U.CARR (IQR: 257–329), respectively]. Oxidative stress values showed no significant correlation with DLCO (%) or FVC (%) in patients. Hydroperoxide concentrations significantly increased in 22 of the 27 patients followed-up for six months. There was no significant difference in baseline oxidative stress parameters between “rapid progressors” (*n* = 13) and “slow progressors” (*n* = 14), but the magnitude of the increase was greater in “rapid progressors” after 6 months. Furthermore, serum hydroperoxide concentrations in the 13 patients with acute exacerbation were significantly higher than those of patients with stable disease [median, 587 U.CARR (IQR: 523–667) vs. 366 U.CARR (IQR: 339–443)]. The authors concluded that, since hydroperoxides in IPF were significantly correlated with lower FVC and acute exacerbation, oxidative stress may be involved not only in IPF development, but also in its progression.

Ugurlu et al. [28] measured total oxidant status (TOS), total antioxidant status (TAS), and oxidative stress index (OSI) in 31 patients with idiopathic interstitial pneumonia (IIP) including 9 patients with IPF, and 33 healthy controls. IIP patients showed higher TOS and OSI values, but similar TAS levels when compared to controls. There was a negative association between TOS and erythrocyte deformability measured at 1.69 Pa, as well as a positive correlation between TAS and erythrocyte deformability measured at 5.33 Pa in IIP patients. These findings suggest that the increased red blood cell aggregation, mediated by oxidative stress, might further disrupt tissue oxygenation in patients with IIP.

Veith et al. [29] measured, in 11 non-smoking patients with IPF and 9 non-smoking healthy controls matched for age, gender and dietary behaviour, blood GSH, GSSG, TEAC, vitamin C and uric acid concentrations. GSH concentrations were approximately 50% lower in IPF patients when compared to controls (6.6 ± 0.6 vs 12.9 ± 1.9 μmol/mg Hb), whereas GSSG concentrations did not significantly differ between the two groups. The total plasma antioxidant capacity in IPF patients was significantly lower when compared to that of controls (483.1 ± 29.5 vs 619.7 ± 16.6 μmol/L Trolox equivalent). The concentrations of uric acid and vitamin C, two endogenous antioxidants known to contribute substantially to the total plasma antioxidant status, were slightly, but not significantly, lower in IPF patients than in controls.

Despite some discrepancy in the results and variability of the methods employed, studies on blood markers reported an increased OS in patients with IPF, which correlates with clinical and functional parameters. Furthermore, OS biomarkers were significantly altered in more severe and rapidly progressive forms and, in some circumstances, predicted the efficacy of inhaled therapy with NAC. This suggests their potential use in the assessment and monitoring of these patients.

### Biomarkers of OS in breath and sputum

The non-invasive measurement of biomarkers in breath or sputum, albeit potentially attractive, is currently limited by issues regarding reproducibility and sensitivity.

Kanoh et al. [30] measured exhaled ethane (a product of lipid peroxidation) in 34 patients with ILD, including 13 with IPF, and 16 control subjects on hospital admission and after three weeks. Exhaled ethane concentrations were significantly higher in ILD patients when compared to controls. Serial measurements revealed that changes of ethane concentrations were associated with disease progress by classifying ILD patients into an IPF group and a non-IPF group. Using an estimated normal range value for exhaled ethane, 4.9 pmol/dL, obtained by adding 2 S.D. values to the mean value in healthy volunteers, the authors found that ethane concentrations were significantly higher in IPF patients. In this group, four patients showed a decrease to less than 5.0 pmol/dL after three weeks of treatment with corticosteroids and immunosuppressive agents. These patients showed no subsequent exacerbations and remained stable. The remaining patients died or experienced significant deterioration.

Beeh et al. [31] measured GSH concentrations in induced sputum of 16 non-smoking IPF patients and 15 healthy subjects. The tGSH concentrations in IPF sufferers was about four-fold lower than that of controls (mean GSH 1.4 ± 0.34 μmol/L vs 5.8 ± 0.98 μmol/L). There was an inverse association between GSH sputum concentrations and disease severity, and a positive correlation between GSH and vital capacity (VC, %), but not DLCO (%).

These studies suggest that the measurement of exhaled ethane or GSH in sputum might be useful in the clinical evaluation and monitoring of patients with IPF. Nevertheless, the evaluation of OS in breath or sputum in these patients warrant further investigations because of the relatively small sample size of published studies and the different OS biomarkers measured with different techniques.

### Biomarkers of OS in exhaled breath condensate

Several mediators have been detected in EBC. However, various factors, highlighted in the recent recommendations by the ERS, may affect their measurement [32]. An important limitation of this approach is the low measured concentrations (often near to the detection limits), which might significantly increase variability. Psathakis et al. [33] measured EBC concentrations of H_2_O_2_ and 8-isoprostane in 16 patients with IPF and 15 healthy subjects. The mean concentration of H_2_O_2_ was significantly higher in patients than in healthy subjects [0.36 μmol/L (95% CI 0.24–0.47) vs. 0.16 μmol/L (95% CI 0.10–0.23)]. The authors observed non-detectable concentrations of H_2_O_2_ in four controls. The mean concentration of 8-isoprostane was also significantly higher in IPF patients compared to controls [74 pg/mL (95% CI 38–110) vs. 33 pg mL (95% CI 28–39)]. There was no significant correlation between H_2_O_2_ and 8-isoprostane concentrations in IPF patients. A negative correlation between H_2_O_2_ and DLCO was observed, however there were no other significant correlations between both markers and pulmonary function tests or pO_2_. Moreover, H_2_O_2_ and 8-isoprostane were not correlated with BALF cell count. In summary, this study evidenced that EBC H_2_O_2_ and 8-isoprostane concentrations are significantly higher in patients with IPF when compared to healthy subjects, and that the concentrations of H_2_O_2_ seem to be associated with disease progression.

Bartoli et al. [34] did not observe significant between-group differences in EBC MDA concentrations in 38 IPF patients and 14 healthy subjects. The authors hypothesized that treatment with oral corticosteroid and/or N-acetyl-cysteine in most IPF patients might have lowered per se EBC MDA concentrations.

Chow et al. [35] collected EBC in 20 IPF patients and 20 controls and measured several oxidative stress and inflammatory biomarkers, including total nitrogen oxides (NOx), 3-nitrosotyrosine (3-NT), 8-isoprostane, total proteins, H_2_O_2_ as exhaled nitric oxide (FeNO) and carbon monoxide (eCO). EBC 3-nitrotyrosine was higher in IPF patients compared to controls [2.5 ng/mL (range 0.7–8.9) vs 0.3 ng/mL (0.1–1.1). 3-NT concentrations significantly correlated with other biomarkers, such as total NOx, 8-isoprostane, total proteins, and H_2_O_2_. Moreover, there was a significant inverse relationship between EBC 3-NT concentrations and FEV1 (%), FVC (%), VC (%), and total lung capacity (TLC, %). No significant between-group differences in total NOx were observed. Furthermore, NOx did not correlate with parameters of lung function. The authors found that IPF patients had significantly higher EBC total protein concentrations than control subjects. Total proteins significantly correlated with 8-isoprostane, H_2_O_2_ and 3-NT, but not with lung function. Mean EBC 8-isoprostane concentrations were also higher in IPF patients when compared to normal subjects [0.2 ng/mL (range 0.1–0.4) vs 0.08 ng/mL (0.04–0.2). Correlation analysis showed significant associations between 8-isoprostane and total proteins, H_2_O_2_, NOx and 3-NT, but not with lung function. There were no between-group differences in EBC H_2_O_2_ concentrations. H_2_O_2_ concentrations were significantly associated with NOx, 3-NT, 8-isoprostane and total protein, but not with lung function.

Finally, Shimizu et al. [36] measured EBC concentrations of the oxidative stress marker 8-iso prostaglandln-F_2α_ (8-isoPGF_2α_) in IPF patients and control subjects. Isoprostanes are prostaglandin (PG)-like substances that are generated through peroxidation of unsaturated fatty acids by free radicals [37]. The isoprostane 8-isoPGF_2α_ is an isomer of PGF1 that is generated by peroxidation of arachidonic acid. 8-isoPGF_2α_ concentrations were significantly higher in IPF patients than control subjects (24.5 ± 15.8 pg/ml vs 4.6 ± 1.0 pg/ml, *p* < 0.05), and suggested that oxidative stress may promote the progression of IPF via ROCK (Rho-associated coiled-coil containing protein kinase) activation. The ROCK pathway is involved in myofibroblast contractility via relaxin (an insulin family peptide) in lungs with IPF and in mice with bleomycin-induced lung fibrosis [38]. ROCK activation may induce contraction of airway and arterial smooth muscle cells, and is involved in angiogenesis impairment [39, 40]; both events have been reported to be involved in the pathogenesis of IPF [41, 42].

### Biomarkers of OS in epithelial lining fluid

One of the first evidence of oxidative stress in IPF was described by Cantin et al. [43] in 1987, who showed that patients with IPF had markedly increased ELF concentrations of myeloperoxidase (MPO). MPO, a member of the XPO subfamily of peroxidases, produces hypochlorous acid (HOCl) from hydrogen peroxide (H_2_O_2_) and chloride anion (Cl^−^) or the equivalent from a non-chlorine halide during the neutrophil’s respiratory burst. Furthermore, it oxidizes tyrosine to tyrosyl radical using hydrogen peroxide. The cytotoxic action of hypochlorous acid and tyrosyl radical impairs antimicrobial capacity of neutrophils [44, 45]. The authors found that the incubation of IPF ELF with cultured alveolar epithelial cells in the presence of H_2_O_2_ caused increased cellular injury, which was suppressed by methionine, a myeloperoxidase system scavenger. The ability of IPF ELF to augment H_2_O_2_-mediated injury to lung parenchymal cells in vitro, additionally, correlated with the ELF concentration of MPO. Moreover, the VC deterioration rate was greater in patients with increased ELF MPO than in those without. These findings suggest that IPF ELF potentially increases epithelial cell cytotoxicity by highly reactive hypohalous anion formation. This toxic radical can oxidize various biomolecules, inducing alterations in critical cellular components and cell death, contributing, at least in part, to the severe epithelial cell injury typical of IPF.

The same authors reported subsequently [46] that the ELF concentration of total glutathione [reduced (GSH) + oxidized (GSSG)] in IPF was significantly lower than that of healthy subjects (97 ± 18 μmol/ vs 429 ± 34 μmol/L). However, ELF glutathione did not correlate with respiratory function tests. Despite differences in the total content of GSH, the ratio of (GSH)/(GSH + GSSG) in patients with IPF was similar to that observed in normal subjects (94 ± 4% vs 98 ± 2%), suggesting that the reduction of GSH in IPF is primarily caused by direct GSH oxidation in the ELF milieu. Starting from the assumption that ELF GSH is derived from epithelial cells, the authors hypothesized that the chronic oxidant burden in IPF causes an alteration in their ability to synthesize, store and secrete GSH. In this regard, studies in neutrophils and lung tissue have shown that OS reduces the intracellular concentrations of both GSH and GSSG [47, 48], and that oxidative stress in general may affect GSH transport in epithelial cells.

The findings of Cantin et al. in ELF GSH were confirmed by two further studies. Borok et al. [49] in 1991 reported lower ELF concentrations of total and reduced GSH in IPF patients when compared to controls. As for the work of Cantin et al. [46] reduced GSH was more than 90% of total ELF glutathione both in IPF sufferers and in controls. IPF subjects were treated with GSH, 600 mg twice daily for 3 days, by aerosol. One hour after the first aerosol the ELF tGSH concentrations were significantly increased. After repeated dosing for 3 days, the average concentration of tGSH was slightly, but not significantly, higher than the pre-therapy concentration, while ELF GSSH increased significantly. A decrease in the spontaneous release of superoxide anion (O_2_^−^) by alveolar macrophages was observed in all subjects.

Concentrations of tGSH in ELF were also measured by Meyer et al. [50], before and after oral therapy with 3 × 600 mg NAC per day for 5 days, in 17 non-smoking patients with IPF. Pre-treatment total glutathione levels in ELF were 187 ± 36 μmol/L, significantly lower than that in normal subjects (368 ± 60 μmol/L). After therapy with NAC, ELF tGSH concentrations increased to 319 ± 92 μmol/L, close to normal values, even if this increase was not statistically significant.

These studies, other than their contribution on the understanding of some of the pathophysiological mechanisms leading to oxidative imbalance in IPF through GSH oxidation, showed that the measurement of GSH in ELF may be useful for the monitoring of OS reduction to specific treatments. Nevertheless, the clinical utility of these responses should be further investigated, because the ELF GSH concentrations were shown not to correlate with respiratory function tests in IPF patients.

### Biomarkers of OS in bronchoalveolar lavage fluid

BALF is a diagnostic procedure assessing cellular and non-cellular components of the epithelial lining fluid of the alveolar and bronchial airspaces for diagnostic, therapeutic, and research purposes. Though BALF is an invasive approach, it is usually well-tolerated and safe in IPF patients [51]. The quantification of biomarkers in the supernatant may be challenging, due to the lack of a satisfactory marker for the dilution of the saline lavage. This is one of the factors that may contribute to the variability in measurements, particularly in studies of limited sample size.

BALF from patients with IPF were analysed by Lenz et al. [52] to evaluate oxidized proteins. The oxidation of proteins, usually accompanied by the introduction of carbonyl groups into their amino acid side-chains, can be measured by labelling these groups with several validated methods [53]. The authors found that carbonyl proteins concentrations were higher in IPF non-smokers when compared to healthy non-smokers (8.3 ± 0.7 vs 5.3 ± 0.6 nmol/mg protein), whereas the IPF smoking group showed decreased levels of carbonyl proteins vs. healthy control smokers (5.9 ± 0.9 vs 8.3 ± 1.5 nmol/mg protein). The total BALF carbonyl protein concentrations in the whole IPF cohort correlated significantly with absolute eosinophil, polymorphonuclear, and neutrophil counts.

The same authors [54] confirmed later that the oxidative status of BALF proteins, defined as carbonyl protein groups, is 2.4-fold higher in IPF. They also described an inverse relationship between the carbonyl content of BALF proteins and glutathione peroxidase mRNA levels, and a positive correlation between oxidized proteins and TNF-α, IL-1b and IL-8 in IPF patients. The authors hypothesized the involvement of an IL-8 dependent mechanism in the marked shift in favour of oxidants in IPF.

Similarly, Rottoli et al. [55] found that IPF patients had significantly higher concentrations of oxidised BALF proteins than controls through a proteomic approach, which revealed that protein carbonylation involved specific carbonylation-sensitive proteins, and that in IPF a greater number of proteic targets of oxidation were present, including albumin, transferrin, α1-antitrypsin, complement C3, SOD, ceruloplasmin, pulmonary surfactant-associated protein A, and many others. The authors confirmed these findings [56] reporting that the protein carbonyl content in IPF BALF of 15 patients were four-fold higher than in controls. In the latter study however, no correlations were found between carbonylated protein concentrations and BALF cell populations, either in the IPF or in the control group.

Again, by means of proteomic differential analysis, Carleo et al. [57] investigated the protein patterns of BALF samples in 10 familial (f-) IPF and 17 sporadic (s-) IPF patients. s-IPF patients showed up-regulation of proteins involved in oxidative stress response, including isocitrate dehydrogenase, peroxiredoxin 1, antithrombin III, and complement factor B. The identified proteins were functionally involved in several biological processes such as ‘cellular iron ion homeostasis’, ‘response to stimulus’, ‘response to stress’, ‘acute inflammatory response’, and others. The authors underlined that differentially expressed proteins in their study are common biomarkers in connective tissue diseases and autoimmune diseases, and this may imply pathophysiological similarities between these conditions and IPF.

Meyer et al. [50] measured BALF GSH concentrations, before and after oral therapy with 3 × 600 mg NAC per day for 5 days, in 17 non-smoking patients with IPF. Pre-treatment tGSH in BALF (0.99 ± 0.12 μmol/L) was similar to controls (1.18 ± 0.19 μmol/L; *p* > 0.5). After therapy with a total of 15 doses of 600 mg NAC, BALF GSH levels significantly increased to 1.54 ± 0.24 μmol/L, within the normal range. The authors emphasised that GSH levels remained high at 10 ± 0.5 h after the last treatment dose.

Rahaman et al. [21] measured several parameters of oxidant/antioxidant balance in BALF of 24 patients with IPF (17 non-smokers and 7 smokers), and 31 healthy subjects (23 non-smokers and 8 smokers). Antioxidant capacity, measured as TEAC, was significantly higher in healthy smokers, compared with healthy non-smokers, while all IPF patients (both smokers and non-smokers) showed lower levels of TEAC when compared to healthy subjects. BALF protein thiols were significantly decreased in non-smoking IPF patients and were similar in IPF smokers compared to either healthy non-smokers or smokers. The authors also found a significant increase in BALF proteins in both groups of patients with IPF compared to healthy individuals. Analysis of GSH levels in BALF in this study revealed that GSH concentrations were significantly lower in IPF non-smokers compared with healthy non-smokers. In contrast, GSH levels were significantly higher in IPF smokers when compared to non-smokers, while no differences were observed with healthy smokers. Evaluation on GSSG levels showed no significant differences in any of the IPF groups when compared to healthy subjects. However, the GSH/GSSG ratio was higher, 73% and 208%, in healthy smokers and smoking IPF patients, respectively, when compared with healthy non-smokers. By contrast, the GSH/GSSG ratio was significantly lower, 64%, in IPF non-smokers compared to healthy non-smokers. In addition, the authors analysed the products of lipid peroxidation in BALF and found that the levels of lipid peroxides were higher in the global IPF cohort when compared to healthy subjects; in particular, MDA levels were significantly higher in patients with IPF who were smokers when compared to healthy smokers.

Markart et al. [58] measured several non-enzymatic low-molecular-weight antioxidants such as GSH, uric acid, ascorbic acid (vitamin C), retinol (vitamin A), and tocopherol (vitamin E) in BALF from 16 patients with IPF. Additionally, they determined BALF plasmalogen levels, the alk-1-enyl-acyl subclass of phosphatidylethanolamine and phosphatidylcholine, a potent antioxidant. The most abundant antioxidant molecule in BAL fluids in the control group was GSH, followed by uric acid and ascorbic acid. The levels of GSH were not significantly altered in IPF patients. By contrast, uric acid levels were significantly higher in IPF patients compared to controls. Similarly, a significant increase in ascorbic acid concentrations was observed in IPF patients (0.87 ± 0.14 μmol/L) vs. controls (0.48 ± 0.04 μmol/L). Moreover, IPF BALF concentrations of both lipophilic antioxidants were strongly increased compared with controls. A significant increase of GSSG, and a trend toward raised levels of F2-isoprostanes, were also reported in IPF BALF. By contrast, plasmalogen concentrations did not significantly differ between cases and controls, and no significant relationships were observed between the concentrations of the studied antioxidants, pulmonary function, and parameters of gas exchange.

Malli et al. [25], other than their studies on blood previously described, compared the levels of 8-isoprostane in BALF of 16 IPF subjects and 55 sarcoidosis patients. Individuals with sarcoidosis had significantly higher 8-isoprostane BALF concentrations when compared to IPF patients [median 220.6 pg/mL (IQR: 133.6–403.3) vs 74.87 pg/mL (IQR. 62.23–115.1), respectively].

The quantification of biomarkers in BALF may be challenging, due to the lack of a satisfactory marker for the dilution of the saline lavage. This is one of the factors that may contribute to the variability in the measurements described, particularly in studies with small sample size. Nevertheless, some studies showing encouraging detection capacity, and previous adequate standardization, might provide alternative options in OS evaluation and monitoring in IPF.

### Biomarkers of OS in lung tissue

Kinnula et al. [59] reported similar extracellular immunoreactivity of superoxide dismutase (ECSOD), the major antioxidant enzyme of the human lung extracellular matrix, in vascular endothelium, bronchial epithelium and alveolar macrophages of 10 patients with biopsy-proven IPF and controls. UIP regenerative alveolar epithelium showed variable ECSOD positivity. Fibrotic lesions and fibroblastic foci were negative for ECSOD. To confirm this finding, the authors excised fibrotic and non-fibrotic areas from OCT-embedded non-fixed frozen sections of UIP lungs to compare ECSOD by western blotting analysis. The findings, in agreement with the immunohistochemical results, confirmed that ECSOD immunoreactivity was significantly lower in fibrotic vs. non-fibrotic areas. The granular cells of the interstitium were the only cells demonstrating intense ECSOD staining in fibrotic areas. The authors hypothesized that the decreased levels of ECSOD in fibrotic areas of UIP may further increase the oxidant burden of the disease.

Vuorinen et al. [60] studied peroxiredoxin (Prx) II, an antioxidant that has been associated with platelet-derived growth factor (PDGF) signalling, and consequent cell proliferation. Localization and expression of Prx II, PDGF receptors (PDGFRa, PDGFRb), Ki67 (a marker of cell proliferation), and nitrotyrosine (a marker of oxidative-nitrosative stress) were assessed in ten IPF/UIP lung biopsies and ten controls by immunohistochemistry and morphometry. The results suggest that Prx II oxidation does not play a significant role in the pathogenesis of IPF/UIP and that Prx II, PDGFRs, and proliferating cells co-localize in the IPF/UIP lung and FF.

Mazur et al. [61] analysed the Nrf2 –sulfiredoxin-1 (SRX1) pathway by several methods to assess the cell-specific localization and expression of NRF2 and SRX1, and selected proteins linked to their activation or stability in human IPF and non-specific interstitial pneumonia (NSIP) patients. Nrf2 induces several antioxidant enzymes [62], including the rate-limiting enzyme in GSH synthesis, gammaglutamyl cysteine ligase, as well as glutathione-S-transferases, thioredoxins, peroxiredoxins, and hemeoxygenase-1. Increased oxidative stress favours the dissociation of the cytoplasmic Kelch-like ECH-associated protein-1 (KEAP1)- NRF2 complex. Consequently, released Nrf2 translocates to the nucleus where it binds to the antioxidant responsive element, and initiates the transcription of the above-mentioned enzymes involved in protection from oxidative stress. Oxidants can also provoke protein and thiol oxidation, nitrosylation and carbonylation. The SRX1 enzyme, regulated by Nrf2, catalyses the reduction of these altered moieties back to their active forms. Non-specific cell variability in the expression of the Nrf2 pathway was observed both in healthy and diseased lungs. By contrast, SRX1 was increased in IPF compared to controls. Immunohistochemistry revealed that proteins of the Nrf2 pathway were localized in the hyperplastic alveolar epithelium and inflammatory cells of IPF lung tissue, but were absent in the fibroblastic foci, which is a characteristic trait of IPF. Morphometric evaluation revealed that Nrf2 and KEAP1 were significantly increased in the hyperplastic alveolar epithelium compared to the normal alveolar epithelium, and Nrf2 was markedly expressed in the nuclear compartment of the hyperplastic cells. SRX1 was expressed mainly in alveolar macrophages, and the number of SRX1-positive macrophages/surface area was elevated in NSIP, a disease with greater inflammatory activity compared to IPF. The authors concluded that the expression of the Nrf2 pathway in human IPF, and NSIP, represents further evidence that the pathogenesis of human fibrotic lung diseases is oxidant-mediated and originates from the alveolar epithelium.

Korfei et al. [63] performed comparative proteomic analysis of peripheral lung tissue from 14 with IPF patients, 8 fibrotic non-specific interstitial pneumonia (fNSIP) and 10 organ donors, by using the 2-dimensional DIGE technique and MALDI-TOF-MS. Downregulated proteins in IPF and fNSIP included antiapoptotic factors and antifibrotic molecules, as well as antioxidant enzymes such as glutathione transferase and haptoglobin. Upregulated proteins included stress-induced molecules involved in the ER stress-pathway, such as leucine aminopeptidase (LAP3) and peptidylprolyl isomerase A (PPIA). The authors found that the IPF and fNSIP proteomic pattern differed only for the expression of a few proteins like the antioxidant acting proteins peroxiredoxin 6 (PRDX6) and thioredoxin peroxidase B (TPxB). The authors advocate that key molecular events in the pathogenesis of IPF and fNSIP are localized in the alveolar epithelium and suggest that antioxidant therapeutic approaches may inhibit detrimental oxidant-mediated reactions, which seem originating mainly from chronic stress.

Finally, a metabolomics approach was used by Kang et al. [64] to characterize metabolic changes of lung tissues involved in the pathogenesis of IPF using gas chromatography−mass spectrometry based metabolic profiling. Analyses were performed on lung tissue samples of 13 patients with IPF and 15 controls. Partial least-squares discriminant analysis (PLS-DA) model generated from metabolite data discriminated between control subjects and IPF patients (receiver operator characteristic area under the curve, AUC > 0.9). In univariate and multivariate statistical analyses twenty-five metabolite signatures of IPF were detected, primarily involving GSH synthesis, including cysteine, glycine, and glutamic acid, found at higher concentration in IPF.

The contribution of studies on IPF tissues was essential for the understanding of several pathogenic mechanisms involving numerous OS protein networks and molecular interplays. The main advantage of lung biopsies is that they directly sample the parenchyma, maintaining the spatial relationships of structural components thus allowing to clearly identify tissue districts in which oxidative stress is present. However, its invasiveness limits patient’s acceptance and justifies its use primarily in situations where the diagnosis remains uncertain with less invasive approaches [48].

## Antioxidants for the treatment of IPF

Considering that oxidative stress is widely recognized as a central feature of IPF, antioxidant therapy has been proposed for many years. In particular, N-acetylcysteine has been widely used in IPF as antioxidant and antifibrotic agent since it is relatively inexpensive, well-tolerated, and easy to administer orally. However, scientific data were often conflictual or inconclusive, mainly due to the lack of placebo arm and the low statistical power.

Two recent papers reported meta-analyses of trials investigating the efficacy of antioxidant therapy in IPF. Sun et al. [65] included 5 trials, with a total of 564 patients, to evaluate the efficacy of NAC in the treatment of IPF. N-Acetylcysteine was found to have a significant beneficial effect on predicted VC and 6 min walking test distance (6 min-WTD), but not on FVC, DLCO, rates of adverse events and mortality. In another meta-analysis, Kandhare et al. [66] identified twelve studies (*n* = 1062) investigating the antioxidants NAC and lecithinized SOD, alone or in combination with other drugs such as pirfenidone, azathioprine, prednisone, to treat IPF. There was no evidence that antioxidant monotherapy had any beneficial effects on changes in predicted DLCO. Combined antioxidant therapy was more effective than monotherapy on VC and DLCO, however this did not translate into differences in death rates or adverse events.

These meta-analyses included the most relevant clinical trials, such as the IFIGENIA and PANORAMA studies [67, 68], which used different methods, control groups, and clinical or functional criteria (rather than biomarkers of OS) in evaluating NAC in IPF. Their findings are in contrast with the results of the PANORAMA study [68], which showed that the NAC plus pirfenidone group had an increased incidence of photosensitivity compared to pirfenidone alone, and a more rapid disease progression, measured as FVC change. This has been discussed by Wijsenbeek and Collard [69] in their comment on the PANORAMA study results, underlying that acetylcysteine therapy cannot be currently recommended to patients with IPF.

Although the 2015 updated treatment guidelines for IPF recommended against NAC [70] NAC monotherapy has been shown to be associated with improved walk distance and mental wellbeing in patients with IPF in other reports [71]. In addition, recent evidence indicates that the response to NAC therapy may be different on the basis of TOLLIP genotype [72]. The gene TOLLIP play important roles in the lung host defence, an immune process influenced by oxidative signalling. In particular, it has been found that NAC may be an effective therapy for individuals with IPF with TOLLIP TT genotype but was associated with a trend toward harm in those with CC genotype. Overall, the therapeutic potential of NAC for patients with IPF remains undefined, and future pharmacogenomic trials must be done to confirm the findings of this study.

## Conclusions

It is currently accepted that IPF pathogenesis depends on repetitive chronic cell injury, which triggers fibrosis and oxidative imbalance within the lung. Causes of oxidative stress include, but are not limited to, cell injury itself, transition metal exposure, inflammation, or drugs that participate in reduction–oxidation reactions. The importance of oxidative modifications to the extracellular matrix, and how they alter cellular responses in human IPF, remains to be elucidated. Since 1987, when the first evidence of an imbalanced oxidant/antioxidant system was described in IPF ELF, several researchers have investigated oxidative stress biomarkers in this pathology. This review summarizes their work on oxidative stress indicators in IPF using different biological sample sources (lung tissue, BALF, EBC, breath, sputum or blood). Although interventional trials failed to demonstrate significant effects of antioxidant treatments (particularly NAC) recent evidence suggest the potential role of pharmacogenomics in predicting efficacy.

## Abbreviations

3-NT: 3-nitrosotyrosine
6 min-WTD: 6 min walking test distance
8-isoPGF_2α_: 8-iso prostaglandln-F_2α_
8-oxodG: 8-oxo-7,8-dihydro-2′-deoxyguanosine
ATS: American thoracic society
BALF: Bronchoalveolar lavage fluid
CAT: Catalase
Cl^−^: Chloride anion
CO: Carbon monoxide
COPD: Chronic obstructive pulmonary disease
DLCO: Diffusing capacity of carbon monoxide
d-ROMs: Derivative ROMs
EBC: Expired breath condensate
ECSOD: Extracellular SOD
ELF: Epithelial lining fluid
ERS: European respiratory society
FEV_1_: Forced Expiratory Volume in the 1st second
FF: Fibroblastic foci
fNSIP: Fibrotic non-specific interstitial pneumonia
FVC: Forced vital capacity
GSH: Glutathione
GSH-Px: Glutathione peroxidase
GSSG: Oxidize glutathione
GST: Glutathione-S-transferase
H_2_O_2_: Hydrogen peroxide
HOCl: Hypochlorous acid
IIP: Idiopathic interstitial pneumonia
IL-1b: Interleukin 1 beta
IL-8: Interleukin 8
ILD: Interstitial lung disease
iNOS: Inducible nitric oxide synthase
IPF: Idiopathic pulmonary fibrosis
KEAP1: Kelch-like ECH-associated protein-1
LAP3: Leucine aminopeptidase
MDA: Malondialdehyde
MPO: Myeloperoxidase
MRC: Medical Research Council
NAC: N-acetylcysteine
NADPH: Nicotinamide adenine dinucleotide phosphate
NO: Nitric oxide
Nrf2: Nuclear factor erythroid-derived 2-like2 protein
NSIP: Non-specific interstitial pneumonia
O2^−^: Superoxide anion
OH^−^: Hydroxyl radical
OSI: Oxidative stress index
PDGF: Platelet-derived growth factor
PON1: Paraoxonase 1
PPIA: Peptidylprolyl isomerase A
PRDX6: Peroxiredoxin 6
Prx: Peroxiredoxin
RNS: Reactive nitrogen species
ROCK: Rho-associated coiled-coil containing protein kinase
ROMs: Reactive oxygen metabolites
ROS: Reactive oxygen species
SOD: Superoxide dismutase
SRX1: Sulfiredoxin-1
TAC: Total antioxidant capacity
TAS: Total antioxidant status
TBA: Thiobarbituric acid
TBARS: Thiobarbituric acid reactive substances
TEAC: Trolox Equivalent Antioxidant Capacity
TLC: Total lung capacity
TNF-α: Tumor necrosis factor alpha
TOS: Total oxidative status
TPxB: Thioredoxin peroxidase B
UCarr: Carratelli units
UIP: Usual interstitial pneumonia
VC: Vital capacity
